# Biological Effects of Advanced Platelet-Rich Fibrin+ (A-PRF+)-Conditioned Medium on Primary Equine Corneal Stromal Cells: A Proof-of-Concept Study

**DOI:** 10.3390/ani16213341

**Published:** 2026-10-23

**Authors:** Maria Rosaria Miranda, Rodolfo Maria Abbate, Mariaelena de Chiara, Barbara Lamagna, Chiara Del Prete, Giuseppe Piegari, Davide De Biase, Chiara Montano, Alessia Bertamino, Pietro Campiglia, Maria Pia Pasolini, Vincenzo Vestuto

**Affiliations:** 1 Department of Pharmacy, University of Salerno, 84084 Fisciano, Italy; 2 Department of Medicine, Case Western Reserve University School of Medicine, Cleveland, OH 44106, USA; 3 Department of Veterinary Medicine and Animal Production, University of Naples Federico II, 80137 Naples, Italy; 4 Department of Human Sciences, Link Campus University, 00165 Roma, Italy; 5 San Rocco Factory, via Montirone 18, 40019 Bologna, Italy

**Keywords:** advanced platelet-rich fibrin (A-PRF+), equine cornea, primary equine corneal stromal cells, regenerative medicine, wound healing, growth factors, collagen type I, platelet concentrates

## Abstract

Corneal wound healing largely depends on the activation of stromal fibroblasts, which coordinate extracellular matrix remodeling and tissue regeneration. Although advanced platelet-rich fibrin (A-PRF+) has emerged as a promising biomaterial in regenerative medicine, its biological effects on equine corneal fibroblasts remain poorly understood. In this study, primary equine corneal fibroblasts were isolated from stromal explants and treated with an A-PRF+-conditioned medium. Cell metabolic activity, viability, intracellular reactive oxygen species, wound healing, cell cycle progression, growth factor secretion, and collagen type I expression were evaluated using colorimetric assays, fluorescence imaging, flow cytometry, and ELISA. A-PRF+ significantly enhanced fibroblast metabolic activity, increased intracellular ROS production, accelerated wound closure and cell cycle re-entry, and stimulated the secretion of PDGF-BB, TGF-β1, and VEGF-A. Furthermore, treatment increased the percentage of collagen type I-positive fibroblasts, supporting enhanced extracellular matrix production. Collectively, these findings demonstrate that A-PRF+ exerts beneficial effects on primary equine corneal fibroblasts by stimulating their metabolic, proliferative, migratory, and secretory activities. These results provide an experimental basis for the use of A-PRF+-based eye drops as a regenerative strategy to improve corneal healing in horses.

## Simple Summary

Corneal injuries are among the leading causes of vision impairment in horses, and effective treatments that promote tissue repair are still limited. Advanced platelet-rich fibrin is a blood-derived biomaterial naturally enriched with molecules that support healing, but its effects on equine corneal fibroblasts have not been fully investigated. In this study, stromal cells isolated from healthy equine corneas were exposed to conditioned medium obtained from allogeneic advanced platelet-rich fibrin plus. The treatment increased cellular metabolic activity, promoted wound closure, stimulated cell proliferation, enhanced the production of collagen type I, and increased the release of growth factors involved in tissue repair. These findings suggest that advanced platelet-rich fibrin may exert beneficial effects on equine corneal fibroblast activity and support processes associated with corneal wound healing, highlighting its potential as a promising biological approach for ocular surface repair.

## 1. Introduction

Corneal wounds and superficial non-healing corneal ulcers are among the most common ophthalmic disorders affecting horses [1]. Corneal healing repair is a highly coordinated biological process involving epithelial cell proliferation and migration, reattachment of the epithelium to the extracellular matrix, and cellular differentiation [2]. Topical administration of autologous and allogeneic blood products, including serum, plasma, and platelet concentrates, has been investigated as adjunctive therapies in horses for the treatment of a variety of corneal disorders to enhance corneal healing [3].

Platelet-rich plasma (PRP), plasma rich in growth factors (PRGF), and platelet-rich fibrin (PRF) are autologous platelet concentrates characterized by higher levels of bioactive molecules, including transforming growth factor (TGF)-β1, epidermal growth factor (EGF), and matrix proteins, compared with serum [4,5,6,7].

Among these products, PRF is a second-generation autologous platelet concentrate obtained by centrifugation of whole blood without anticoagulants, resulting in a three-dimensional fibrin matrix [8,9]. Within the fibrin scaffold, platelets gradually release growth factors, whereas leukocytes contribute to modulating inflammation and secrete cytokines that support tissue repair and regeneration [10,11]. Advanced formulations such as advanced platelet-rich fibrin (A-PRF) and advanced platelet-rich fibrin plus (A-PRF+), produced using modified low-speed centrifugation protocols, increase the retention of platelets, leukocytes, and bioactive molecules within the fibrin matrix, with A-PRF+ providing a higher cellular content than conventional A-PRF, thereby resulting in prolonged growth factor release and improved regenerative potential [12,13].

These blood derivatives have been evaluated both in vitro and in vivo for their effects on equine corneal cells and corneal wound healing in horses and donkeys. However, the available evidence remains limited, with contradictory findings [14,15,16,17].

Clinical evidence supporting the use of platelet concentrates for corneal disease is increasing, although results remain inconsistent. In humans, small animals, and donkeys, topical PRP has proven to significantly accelerate corneal epithelial wound healing compared with phosphate-buffered saline or autologous serum [18,19,20,21,22]. Nevertheless, no controlled clinical studies have been performed on horses, and current evidence is still insufficient to recommend PRP as standard care due to methodological limitations. Topical application of standard PRF (s-PRF) eye drops in donkeys [10] and PRF gel in rabbits failed to significantly improve experimental corneal wound healing, whereas A-PRF gel effectively promoted corneal healing and controlled ocular surface infection in experimentally induced corneal ulcers in donkeys [9]. The histological evaluation demonstrated that the PRF gel group had reduced inflammation, improved re-epithelialization, absence of epithelial keratinization, and superior collagen organization and maturation compared with untreated controls. Furthermore, PRF membranes have been successfully used as grafting material for reconstruction of corneal ulcers in dogs [9,23], while combined PRF clots and injectable PRF have shown encouraging preliminary results in horses with ulcerative keratitis [11].

In vitro investigations have also demonstrated the regenerative potential of PRGF, which has been found to stimulate scarless regeneration of the ocular surface [2,24]. On the other hand, a comparative in vitro study showed that proliferation of corneal and limbal cells remained constant in the PRGF group and decreased upon serum treatment; meanwhile, in the PRP group the best results were observed, due to the beneficial effects on both proliferation and migration capacity of equine corneal cells [8].

These findings may suggest that platelet-derived products exert different biological effects depending on their composition and preparation protocol. Indeed, variations in manufacturing procedures and study design may substantially influence both experimental outcomes and clinical efficacy.

Building upon our previous findings demonstrating the regenerative potential of equine A-PRF+ in primary skin-derived fibroblasts [25], the present study extends this investigation to primary equine corneal fibroblasts. By exploring the biological activity of A-PRF+ in distinct tissue-specific cell models, we aim to broaden its potential regenerative applications while further elucidating the biochemical mechanisms underlying its pro-regenerative effects.

Therefore, the aim of this study was to investigate the in vitro pro-regenerative effects of an equine A-PRF+-conditioned medium on primary ECSCs. Cellular metabolic activity, migratory capacity, cell cycle progression, and intracellular ROS levels were evaluated. In addition, the secretion of selected growth factors and the expression of collagen type I were assessed to investigate potential effects on extracellular matrix remodeling and the cellular biochemical response.

## 2. Materials and Methods

### 2.1. Animals and Sample Collection

To obtain ECSCs, equine eyes were immediately enucleated from one horse, aged 18 years, and a foal, aged 5 days, which had been euthanized because of severe orthopedic diseases unrelated to this study. Prior to euthanasia, a complete ophthalmic examination was performed by an ophthalmologist with more than 20 years of experience (BL), on both equine eyes, including neuro-ophthalmological examination, STT-1 (Dina strip, DYASET s.r.l., Portomaggiore, FE, Italy), slit-lamp biomicroscopy (Kowa SL-15, Kowa Company Ltd., Nagoya, Japan), direct ophthalmoscopy (Panoptic ophthalmoscope, Welch Allyn, Skaneateles Falls, NY, USA) and rebound tonometry (TonoVet, Icare Finland Oy, Vantaa, Finland). Fluorescein testing, mydriatics, and topical anesthesia were not applied to reduce the cytotoxic effects on the corneal epithelium [8,26]. Prior to enucleation, the globes were rinsed with 0.3% tobramycin eye drops (Stilbiotic, 3 mg/mL, CEVA, Milan, Italy). After enucleation, globes were placed in 1% povidone iodine solution for 20 min and then rinsed with saline. The whole cornea was removed under sterile conditions, working under a hood, within 1 h post-mortem using a #15 Bard Parker scalpel blade. In total, 2–3 mm corneal stromal explants were collected using a #15 scalpel blade and a 0.3 mm Colibri. The corneas, which did not show any macroscopic abnormalities, were sectioned into two pieces and placed in a 50 mL Falcon tube (352,070, Fisher Scientific Oy—Ratastie 201,620 Vantaa—Finland) in sterile PBS solution, supplemented with penicillin/streptomycin 0.1% and kanamycin 0.1%, and transported to the laboratory within 1 h, similarly to what was reported by Rushton and colleagues [8].

### 2.2. Preparation of A-PRF+

Whole blood was collected from the left jugular vein of 5 healthy horses during routine blood sampling procedures, using a Vacutainer blood collection system (BD Vacutainer Safety-Lok Blood Collection Set, 21G × 3/4″ × 7″; Becton Dickinson and Company, Franklin Lakes, NJ, USA). Eight sterile 10 mL glass tubes without anticoagulant (A-PRF tubes, Process for PRF™, Nice, France; Mectron, Cologne, Germany) were collected in total, with one or two A-PRF tubes added to the tubes used for routine clinical analyses while the needle remained in the jugular vein. Thus, no additional venipuncture was required in either horse. Within 2 min of collection, the tubes were centrifuged using a horizontal centrifuge (ALC 4218 Centrifuge, ALC International, Cologno Monzese, Milan, Italy) at 200× *g* for 8 min to obtain A-PRF+, as previously described [17]. After centrifugation, the tubes were stored in a portable cooler at 4 °C and transported to the laboratory within 1 h for subsequent processing.

### 2.3. Preparation of an A-PRF+-Conditioned Medium

An A-PRF+-conditioned medium was prepared from A-PRF+ clots as previously described [25]. Briefly, each clot was transferred into a sterile 50 mL polypropylene conical tube containing 10 mL of serum-free DMEM and incubated at 37 °C for 3 days under gentle agitation using a plate shaker. At the end of the incubation period, the conditioned medium was collected and centrifuged at 100× *g* for 5 min to remove residual debris. The resulting supernatants were pooled, aliquoted, and stored at −80 °C until use. Control medium was processed under the same conditions to ensure consistency among experimental groups. For all in vitro experiments, an A-PRF+-conditioned medium was diluted 1:4 in serum-free DMEM before use. Cells cultured in serum-free DMEM and DMEM supplemented with 10% FBS served as negative and positive controls, respectively.

### 2.4. Establishment and Propagation of Primary Equine Corneal Fibroblasts

Primary equine corneal fibroblasts from the two previously described horses were established from corneal stromal tissue using an explant culture method adapted from the protocol described by Netto et al. [27]. Briefly, corneas were processed under sterile conditions and repeatedly washed with sterile PBS to remove residual blood and debris. The corneal epithelium and endothelium were carefully removed, and the remaining stromal tissue was cut into small fragments of approximately 1–2 mm^3^ using sterile scalpels.

The tissue explants were placed in 6-well culture plates (Corning, Corning, NY, USA) and cultured in Dulbecco’s Modified Eagle Medium (DMEM) supplemented with 10% fetal bovine serum (FBS), 2 mM L-glutamine, 100 U/mL penicillin, 0.1 mg/mL streptomycin, and 2.5 μg/mL amphotericin B. Cultures were maintained at 37 °C in a humidified atmosphere containing 5% CO_2_. The culture medium was carefully replaced every 3 days without disturbing the tissue fragments.

After approximately 2 weeks, spindle-shaped fibroblasts migrated from the corneal stromal explants and adhered to the culture surface. The tissue fragments were then removed, and ECSCs were detached using 0.5% trypsin and 0.2% EDTA, transferred to culture dishes, and expanded under the same culture conditions up to passage 2, at which point cells were cryopreserved in a freezing solution consisting of 90% FBS and 10% DMSO. For subsequent experiments, cells were thawed and used between passages 3 and 6. Cell morphology and viability were routinely monitored by phase-contrast microscopy and trypan blue exclusion assay [28]. All treatments and analyses were performed using primary ECSCs derived from two independent horses. For each donor, experiments were performed using three different cell passages on separate days, yielding six experimental replicates in total. Technical replicate wells were averaged for each treatment within each experimental run. The resulting six experimental values were used to calculate the mean and standard deviation reported in the graphs. In each experiment, cells were exposed to the pooled allogeneic A-PRF+-conditioned medium under the same experimental conditions.

### 2.5. Phenotypic Characterization of Primary Equine Corneal Fibroblasts

ECSCs (5 × 10^4^ cells/well) were seeded into 24-well plates and allowed to adhere for 24 h. Vimentin expression was assessed as a marker of the mesenchymal phenotype. Cells were then washed with PBS and incubated with BioTracker™ TiY Vimentin Live Cell Dye [29] (Sigma-Aldrich, St. Louis, MO, USA) diluted in phenol red-free, serum-free DMEM at a final concentration of 2.5 μg/mL for 20 min at 37 °C in the dark. Nuclei were counterstained with 1.6 μM Hoechst 33342 (Sigma-Aldrich, St. Louis, MO, USA). After staining, cells were washed with PBS, and representative images were acquired using a ZOE Fluorescent Cell Imaging System (20× magnification; scale bars: 100 μm; N  ≥  3).

For quantitative analysis, cells were detached, resuspended in PBS containing 2% BSA, incubated with BioTracker™ TiY Vimentin Live Cell Dye (2.5 μg/mL) for 20 min at 37 °C in the dark, and then analyzed using a FACScan flow cytometer (Becton Dickinson, Franklin Lakes, NJ, USA) equipped with CellQuest software (Version 4).

### 2.6. Cellular Metabolic Activity Assay

Cell metabolic activity was evaluated by assessing mitochondrial dehydrogenase activity using the MTT (3-(4,5-Dimethylthiazol-2-yl)-2,5-diphenyltetrazolium bromide) assay [30]. ECSCs (1 × 10^4^ cells/well) were seeded into 96-well plates and allowed to adhere for 24 h before treatment with an A-PRF+-conditioned medium for an additional 24 h. Subsequently, MTT solution was added to a final concentration of 0.5 mg/mL, and cells were incubated for 4 h. Absorbance was measured at 570 nm using a microplate reader (Multiskan Go, Thermo Scientific, Waltham, MA, USA). Cell metabolic activity was expressed as a percentage relative to the untreated cells cultured in serum-free DMEM and set to 100% metabolic activity, whereas 10% DMSO was used as a negative control and set to 0% of metabolic activity. Cells treated with medium containing 10% FBS were used as a positive control for metabolic activity.

### 2.7. Live-Dead Cell Staining

ECSCs (1 × 10^4^ cells/well) were seeded into 96-well plates and allowed to adhere for 24 h. Cells were then incubated with an A-PRF+-conditioned medium for an additional 24 h. Serum-free DMEM and DMEM supplemented with 10% FBS were used as negative and positive controls, respectively. After treatment, cells were washed with PBS and incubated with 3 μM Calcein-AM and 15 μM PI for 20 min at 37 °C in the dark. Following staining, cells were washed with PBS, and representative fluorescence images were acquired using a ZOE Fluorescent Cell Imaging System based on a methodology previously described [31] (20× magnification; scale bars: 100 μm).

### 2.8. ROS Quantification

ROS levels were measured using 20 μM DCFH-DA (Sigma-Aldrich, St. Louis, MO, USA). ECSCs (1 × 10^4^ cells/well) were seeded in 96-well plates and allowed to adhere for 24 h. Cells were then incubated with an A-PRF+-conditioned medium for 24 h. Serum-free DMEM and DMEM supplemented with 10% FBS were used as negative and positive controls, respectively. After treatment, cells were washed with PBS and incubated with DCFH-DA diluted in phenol red-free, serum-free DMEM for 20 min at 37 °C in the dark. Following staining, the cells were washed, and representative images were acquired using a ZOE Fluorescent Cell Imaging System (20× magnification. Scale bars: 100 μm. N  ≥  3). Fluorescence intensity (excitation/emission: 485/535 nm) was measured using an EnSpire multimode plate reader (PerkinElmer, Waltham, MA, USA) and expressed as DCFH-DA fluorescence intensity (a.u.) [32].

### 2.9. Cell Cycle Analysis

ECSCs (5 × 10^4^ cells/well) were seeded into 24-well plates and allowed to adhere for 24 h. Cells were treated with an A-PRF+-conditioned medium, serum-free DMEM, or 10% FBS-supplemented DMEM, which served as the experimental condition, negative control, and positive control, respectively. After 72 h, cells were collected by trypsinization, centrifuged, and resuspended in a hypotonic staining solution containing 50 µg/mL propidium iodide, 0.1% sodium citrate, and 0.1% Triton X-100. Samples were incubated for 30 min at 4 °C and analyzed using a FACScan flow cytometer (Becton Dickinson) equipped with CellQuest software. Cellular debris and doublets were excluded from the analysis, and the percentages of cells in the G0/G1, S, and G2 phases were calculated according to DNA content [33].

### 2.10. Wound Healing Assay

For the wound healing assay, ECSCs (6 × 10^5^ cells/well) were seeded into 24-well plates and cultured for 24 h at 37 °C to allow the formation of a confluent monolayer. A linear wound was then generated in the center of each well using a sterile 10 μL pipette tip. Cell debris was removed by washing with PBS, and a fresh A-PRF+-conditioned medium was added. Serum-free DMEM and DMEM supplemented with 10% FBS were used as negative and positive controls, respectively. Wound closure was monitored by phase-contrast microscopy, and representative images were acquired immediately after scratching (0 h) and after 72 h of treatment. The wound area was quantified using ImageJ software (version 1.47) and expressed as the percentage of the remaining wound area relative to time 0 [34].

### 2.11. Determination of Collagen Type I Production

ECSCs (5 × 10^4^ cells/well) were seeded into 24-well plates and allowed to adhere for 24 h. Cells were then treated with an A-PRF+-conditioned medium for 24 h. Serum-free DMEM and DMEM supplemented with 10% FBS were used as negative and positive controls, respectively, while unstained cells were included as the unstained control. After treatment, cells were collected, fixed in fixing buffer (2% BSA and 1% formaldehyde in PBS) for 20 min, and centrifuged at 600× *g* for 10 min. The cell pellet was resuspended in permeabilization buffer (2% BSA, 1% formaldehyde, and 0.1% Triton X-100 in PBS) containing the mouse anti-collagen type I primary antibody (Novus Biologicals, Bio-Techne, Minneapolis, MN, USA) and incubated for 30 min. Afterward, cells were incubated with Alexa Fluor 488-conjugated anti-mouse secondary antibody (Molecular Probes, Invitrogen, Waltham, MA, USA), washed again, and finally resuspended in 200 μL of fixing buffer [35]. Samples were analyzed using a FACScan flow cytometer (Becton Dickinson, Franklin Lakes, NJ, USA) equipped with CellQuest software (Version 4). The results were expressed as the percentage of collagen type I-positive cells.

### 2.12. Quantification of Growth Factors Release

ECSCs (5 × 10^4^ cells/well) were seeded into 24-well plates and allowed to adhere for 24 h. Cells were then incubated with an A-PRF+-conditioned medium for an additional 24 h. Serum-free DMEM and DMEM supplemented with 10% FBS were used as negative and positive controls, respectively. At the end of the treatment, culture supernatants were collected and centrifuged at 500× *g* for 5 min to remove cellular debris. The clarified supernatants were used for the quantitative determination of TGF-β1, VEGF-A, and PDGF-BB using commercially available equine ELISA kits (MyBioSource, Inc., San Diego, CA, USA), according to the manufacturer’s instructions. Baseline concentrations of TGF-β1, VEGF-A, and PDGF-BB were also measured in an uncultured 1:4 diluted A-PRF+-conditioned medium and subtracted from the corresponding concentrations measured in the supernatants of A-PRF+-treated cells.

### 2.13. Statistical Analysis

Statistical analyses were performed using GraphPad Prism version 8.0 (GraphPad Software, San Diego, CA, USA). Data normality was assessed using the Shapiro–Wilk test. Differences among groups were analyzed by one-way analysis of variance (ANOVA), followed by Bonferroni’s post hoc multiple-comparison test. Differences were considered statistically significant when *p* < 0.05.

## 3. Results

### 3.1. Isolation and Characterization of Primary Equine Corneal Fibroblast-Like Cells

**Figure 1:**
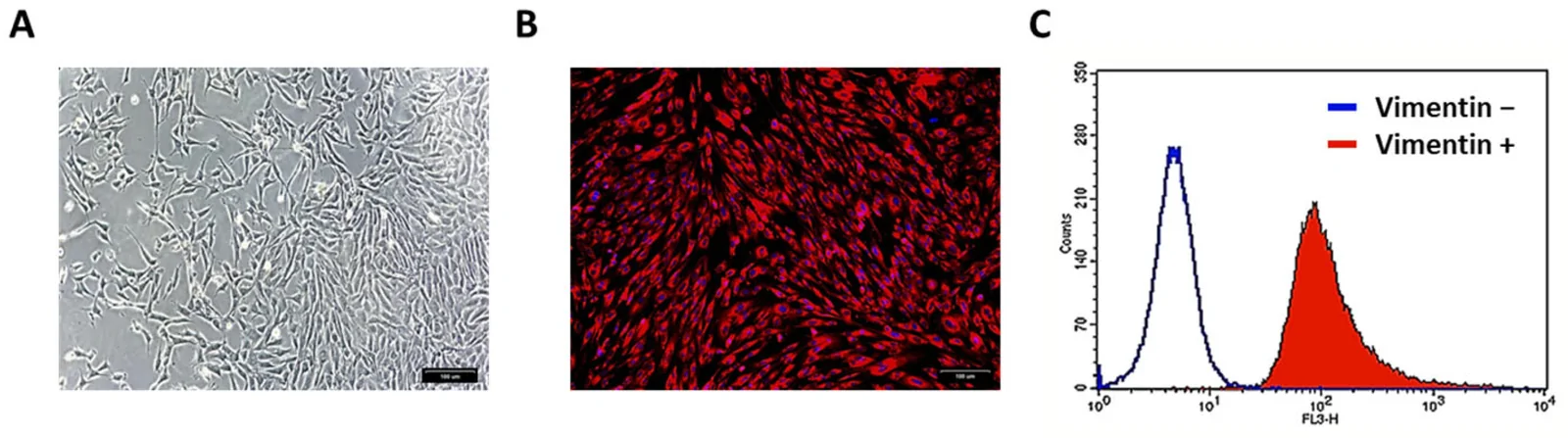
Morphological and phenotypic characterization of primary equine corneal stromal cells. (**A**) Representative phase-contrast image showing the typical spindle-shaped morphology of primary equine corneal fibroblasts. (**B**) Fluorescence microscopy of cells stained with BioTracker™ TiY Vimentin Live Cell Dye (red) and Hoechst 33342 for nuclei (blue). (**C**) Representative flow cytometric analysis showing a vimentin-positive cell population. Scale bar: 100 μm.

Approximately two weeks after corneal explant plating, adherent cells progressively migrated from the stromal tissue fragments and formed colonies around the explants. Phase-contrast microscopy showed cells with the typical elongated, spindle-shaped morphology and flattened appearance characteristic of fibroblast-like cells (Figure 1A).

The cellular phenotype was further evaluated using BioTracker™ TiY Vimentin Live Cell Dye, while nuclei were counterstained with Hoechst 33342 (Figure 1B). Fluorescence microscopy revealed a clear cytoplasmic vimentin signal distributed throughout the cells, supporting their mesenchymal and fibroblast-like phenotype. Flow cytometric analysis further confirmed a homogeneous vimentin-positive population, with essentially all gated cells expressing vimentin (Figure 1C).

### 3.2. A-PRF+ Promotes Cell Metabolic Activity and Intracellular ROS Production

**Figure 2:**
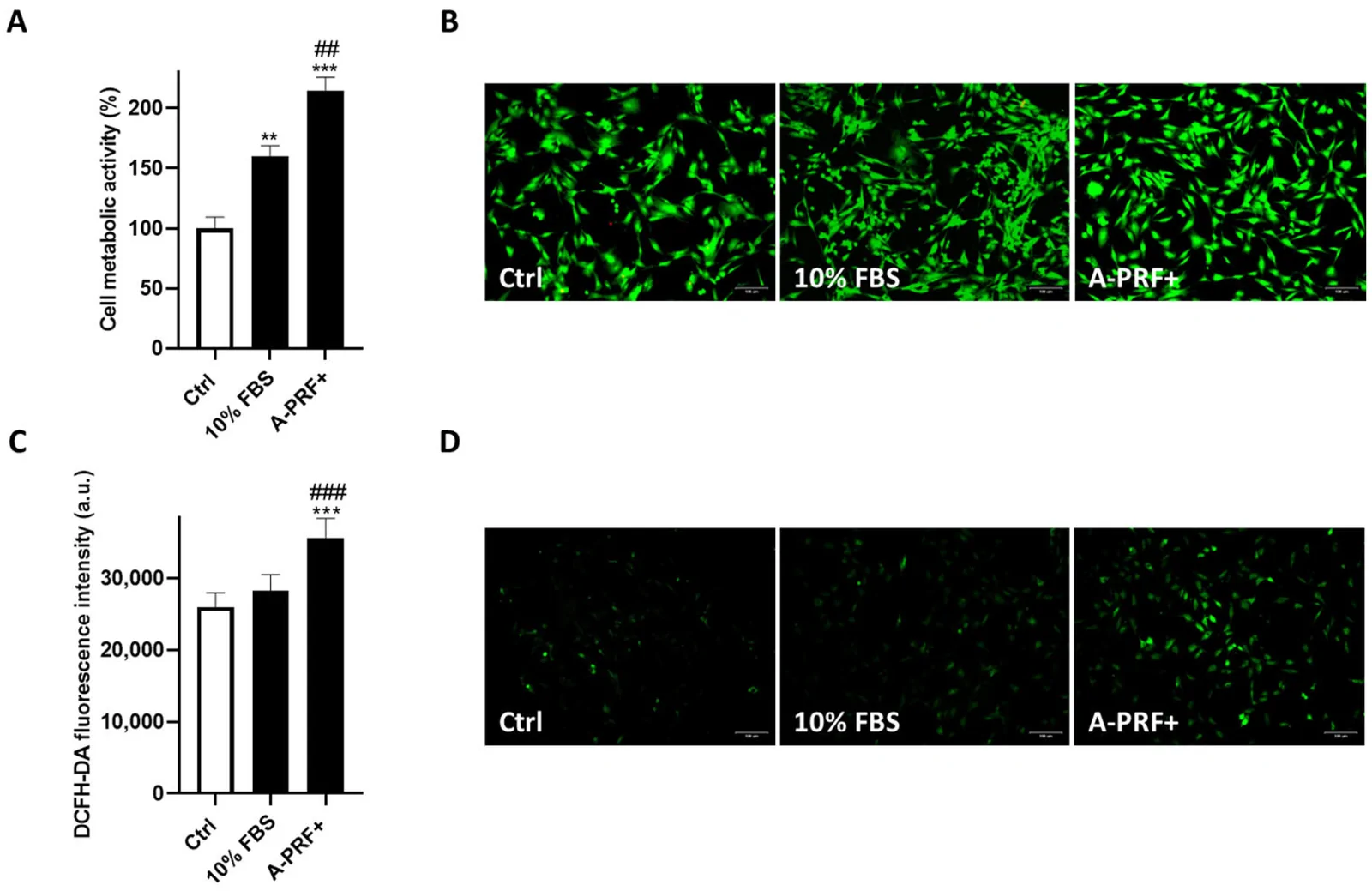
A-PRF + improves cell metabolic activity and ROS production of primary equine fibroblasts. (**A**) Cell metabolic activity was assessed by the MTT assay after 24 h of treatment. (**B**) Representative fluorescence images of Calcein-AM/PI staining after 24 h of treatment. (**C**) Quantitative analysis of intracellular ROS production after 24 h of treatment. (**D**) Representative fluorescence images of ROS-positive cells stained with DCFH-DA after 24 h of treatment. Magnification: 20×. Scale bars: 100 μm. Data are presented as mean ± standard deviation (SD) from six experimental replicates obtained using three cell passages from each of two independent horses. Technical replicate wells were averaged within each experimental run. ** *p* < 0.01 and *** *p* < 0.001 vs. Ctrl; ## *p* < 0.01 and ### *p* < 0.001 vs. 10% FBS.

The effect of an A-PRF+-conditioned medium on primary equine corneal fibroblast metabolic activity was evaluated after 24 h by the MTT assay (Figure 2A). As expected, cells cultured in DMEM supplemented with 10% FBS showed a significant increase in metabolic activity compared with the serum-free control (160.02 ± 8.61% vs. 100.00 ± 9.36%, *p* < 0.01 vs. Ctrl).

Treatment with an A-PRF+-conditioned medium further enhanced fibroblast metabolic activity, reaching 214.32 ± 11.25% of the serum-free control, representing a significant increase compared with both the Ctrl group (*p* < 0.001) and the 10% FBS group (*p* < 0.01). These findings were supported by Calcein-AM/PI live-cell staining, which showed a higher number of viable active cells in the A-PRF+ group compared with the control group, whereas no appreciable propidium iodide-positive cells were observed (Figure 2B and Figure S1).

Since cellular metabolic activation is frequently associated with changes in intracellular redox status, ROS production was subsequently evaluated by DCFH-DA staining (Figure 2C and Figure S2). No significant differences were detected between fibroblasts cultured in serum-free DMEM and those maintained in DMEM supplemented with 10% FBS (26,011.40 ± 1950.82 vs. 28,271.20 ± 2224.66 a.u., *p* = 0.2985 vs. Ctrl).

In contrast, treatment with an A-PRF+-conditioned medium significantly increased intracellular ROS levels, rising to 35,606.40 ± 2715.21 a.u., compared with both the Ctrl group (*p* < 0.001) and the 10% FBS group (*p* < 0.001). Representative fluorescence images were consistent with the quantitative analysis, showing a stronger DCFH fluorescence signal in A-PRF+-treated fibroblasts (Figure 2D).

### 3.3. A-PRF+ Promotes In Vitro Wound Closure and Cell Cycle Re-Entry

The effects of an A-PRF+-conditioned medium on primary equine corneal fibroblast function were further investigated by evaluating cell migration and in vitro wound closure.

**Figure 3:**
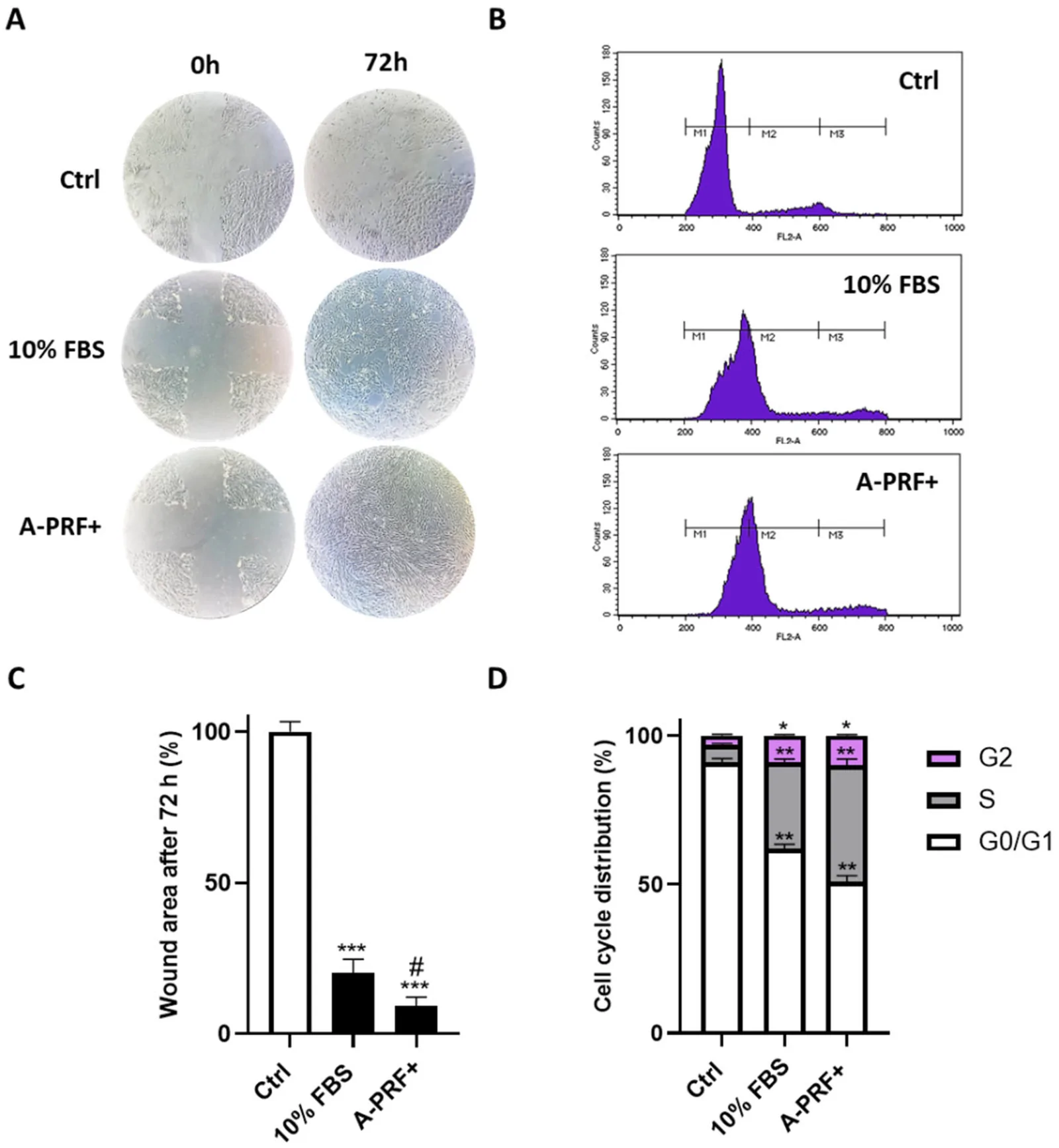
A-PRF+-conditioned medium enhances the wound-healing properties of primary equine corneal fibroblasts and modulates the cell cycle. (**A**) Representative phase-contrast images of the wound healing assay at 0 and 72 h. (**B**) Representative DNA content histograms obtained by propidium iodide staining for cell cycle analysis. M1 = G0/G1 phase positive cells area; M2 = S phase positive cells area; M3 = G2 phase positive cells area. (**C**) Quantitative analysis of the remaining wound area after 72 h. (**D**) Quantitative distribution of fibroblasts in the G0/G1, S, and G2 phases of the cell cycle. Data are presented as mean ± standard deviation (SD) from six experimental replicates obtained using three cell passages from each of two independent horses. Technical replicate wells were averaged within each experimental run. * *p* < 0.05, ** *p* < 0.01, and *** *p* < 0.001 vs. Ctrl; # *p* < 0.05 vs. 10% FBS.

Consistent with the metabolic findings, A-PRF+ markedly enhanced wound closure, as demonstrated by the wound healing assay (Figure 3A). After 72 h, serum-starved cells retained almost the entire wound area and were used as the reference (100.00 ± 3.31%). In contrast, fibroblasts cultured in DMEM supplemented with 10% FBS displayed a marked reduction in the residual wound area (20.17 ± 4.58%, *p* < 0.001 vs. Ctrl). An even greater effect was observed following A-PRF+ treatment, with the residual wound area decreasing to 9.35 ± 2.82%, significantly lower than both the serum-free control (*p* < 0.001) and the 10% FBS group (*p* < 0.05) (Figure 3C).

Cell cycle analysis further supported the previous results obtained by A-PRF+ treatment (Figure 3B). Serum starvation for 24 h induced a marked accumulation of Ctrl cells in the G0/G1 phase, consistent with cell cycle arrest and the maintenance of a quiescent state. As expected, stimulation with 10% FBS significantly reduced the proportion of cells in G0/G1 (*p* < 0.01 vs. Ctrl), with a concomitant increase in the S (*p* < 0.01 vs. Ctrl) and G2 (*p* < 0.05 vs. Ctrl) phases, indicating increased cell-cycle progression compared to Ctrl. A comparable redistribution was observed following A-PRF+ treatment (G0/G1: *p* < 0.01 vs. Ctrl; S: *p* < 0.01 vs. Ctrl; G2: *p* < 0.05 vs. Ctrl), which promoted the transition from quiescence to the proliferative phases of the cell cycle, confirming its ability to stimulate cell cycle progression in primary equine corneal fibroblasts (Figure 3D).

### 3.4. A-PRF+ Stimulates Growth Factor Secretion and Collagen Type I Expression

To better characterize the regenerative response of primary equine corneal fibroblasts, the secretion of key growth factors was quantified by ELISA, while intracellular collagen type I expression was evaluated by flow cytometry.

**Figure 4:**
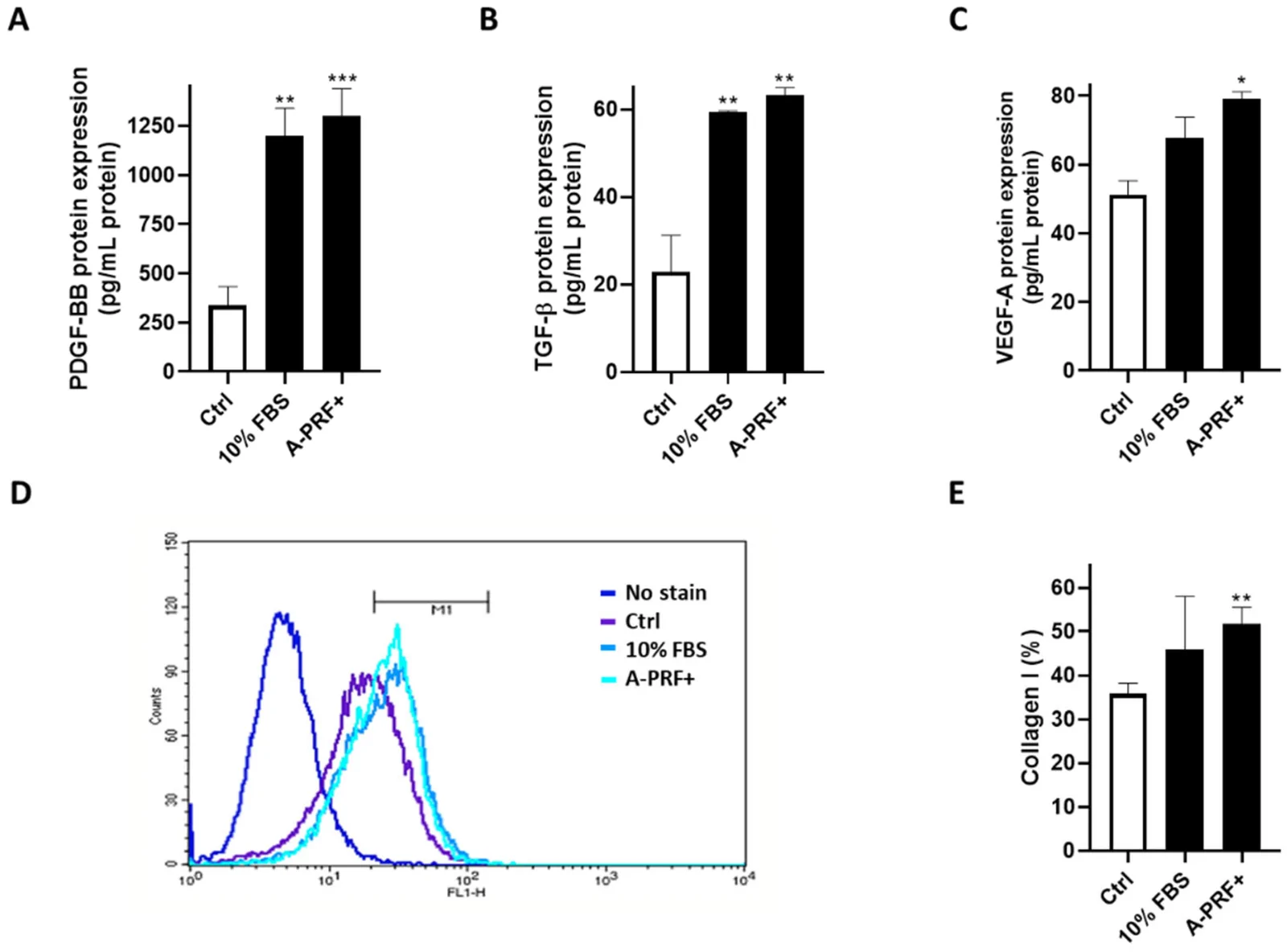
A-PRF+ promotes the expression and release of pro-regenerative factors in primary equine corneal fibroblasts. (**A**) Quantitative determination of PDGF-BB release by ELISA after 24 h of treatment. (**B**) Quantitative determination of TGF-β1 release by ELISA after 24 h of treatment. (**C**) Quantitative determination of VEGF-A release by ELISA after 24 h of treatment. (**D**) Representative flow cytometry histograms showing intracellular collagen type I staining in unstained cells, serum-free Ctrl, 10% FBS, and A-PRF+-treated fibroblasts after 24 h of treatment. M1 = Collagen I-positive cell area. (**E**) Quantitative analysis of collagen type I-positive fibroblasts determined by flow cytometry. Growth factor concentrations are expressed as baseline-corrected values, calculated by subtracting the corresponding baseline concentrations measured in the culture medium. Data are presented as mean ± standard deviation (SD) from six experimental replicates obtained using three cell passages from each of two independent horses. Technical replicate wells were averaged within each experimental run. * *p* < 0.05, ** *p* < 0.01, and *** *p* < 0.001 vs. Ctrl.

ELISA analysis demonstrated that treatment with an A-PRF+-conditioned medium enhanced the release of growth factors associated with tissue repair. In particular, PDGF-BB secretion increased from 337.7 ± 93.7 pg/mL in serum-free controls to 1298.0 ± 140.5 pg/mL (*p* < 0.01), whereas TGF-β1 levels increased from 22.9 ± 8.3 pg/mL to 63.3 ± 1.6 pg/mL (*p* < 0.01). A significant increase was also observed for VEGF-A, which rose from 51.3 ± 4.0 pg/mL in control cells to 79.1 ± 2.0 pg/mL following A-PRF+ treatment (*p* < 0.05) (Figure 4A–C). Baseline levels measured in an uncultured 1:4 diluted A-PRF+-conditioned medium were 139.1 ± 93.7 pg/mL for PDGF-BB, 3.25 ± 0.1 pg/mL for TGF-β1, and 1.7 ± 0.1 pg/mL for VEGF-A, and were significantly lower than the corresponding levels measured in A-PRF+-treated ECSC (*p* < 0.001 for all comparisons) (Table S1).

Flow cytometric analysis further showed that A-PRF+ increased intracellular collagen type I expression. Representative histograms revealed a rightward shift in the fluorescence peak in A-PRF+-treated fibroblasts compared with the unstained and serum-free controls, indicating a greater proportion of collagen type I-positive cells (Figure 4D). Quantitative analysis confirmed this observation, with collagen type I-positive cells increasing from 35.8 ± 2.4% in serum-free controls to 51.8 ± 3.8% following A-PRF+ treatment (*p* < 0.01) (Figure 4E).

## 4. Discussion

The present study investigated the biochemical response of primary equine corneal fibroblasts to conditioned medium derived from A-PRF+. Given the exploratory, proof-of-concept nature of the study, A-PRF+-conditioned medium obtained from five donor horses was pooled to provide a common preparation for the preliminary assessment of its biological effects, rather than to investigate inter-individual variability in A-PRF+ composition or activity.

Overall, the findings indicate that A-PRF+ promotes a coordinated cellular response involving increased metabolic activity, in vitro wound closure, cell-cycle progression, growth factor release, and collagen type I expression. These results both confirm and extend our previous observations in equine fibroblasts isolated from skin biopsies. In our previous study, A-PRF+ enhanced fibroblast metabolic activity, proliferation, and migration, promoted cell cycle re-entry, and increased the production of growth factors and collagen type I. These effects were associated with increased ROS production and adaptive changes in mitochondrial, endoplasmic reticulum, and lysosomal activity, with ROS signaling playing a central role in the cellular response to A-PRF+ [25]. The present study shows that several of these responses are also conserved in corneal stromal cells. However, unlike our previous study, which primarily investigated ROS-dependent metabolic and organelle responses and proteomic remodeling in dermal fibroblasts, the present work extends the biological relevance of A-PRF+ to the corneal stromal context. This distinction is particularly relevant because corneal stromal cell activation and extracellular matrix remodeling are essential components of corneal wound repair, while their dysregulation may contribute to stromal scarring and impaired corneal transparency. Thus, these results provide tissue-specific information relevant to corneal wound healing beyond the general stimulatory effects of A-PRF+ on fibroblasts. Primary cells were obtained from corneal stromal explants; the outgrowing ECSCs displayed an elongated, spindle-shaped morphology and were positive for vimentin, supporting their mesenchymal and fibroblast-like phenotype. Vimentin is an intermediate filament protein widely expressed in mesenchymal cells and is commonly used to characterize cultured fibroblasts, including corneal stromal fibroblasts. Equine corneal keratocytes, fibroblasts, and myofibroblasts can all be isolated and propagated in vitro, and cultured corneal fibroblasts typically show vimentin expression [36,37]. Nevertheless, vimentin is not a fibroblast-specific marker and may also be expressed by other mesenchymal cell populations. Therefore, although the combination of stromal origin, explant-derived growth, spindle-shaped morphology, and vimentin positivity supports a mesenchymal, fibroblast-like phenotype, these features do not allow definitive discrimination among keratocytes, activated fibroblasts, and myofibroblasts. This limitation is particularly relevant in the cornea, where resident keratocytes can undergo a transition toward activated fibroblast and myofibroblast phenotypes in response to serum and growth factors [38,39].

The first response evaluated after A-PRF+ exposure was cellular metabolic activity. After 24 h, the A-PRF+-conditioned medium markedly increased cell metabolic activity compared with both serum-free and 10% FBS controls. This increase was accompanied by a high number of calcein-positive viable cells and the absence of appreciable propidium iodide-positive nuclei.

These findings suggest that the increased signal reflects cellular oxidoreductase activity and may therefore indicate enhanced early metabolic activity in mitochondria, but also in the cytoplasmic membrane and other organelles following exposure to soluble components released from A-PRF+ [40,41].

This metabolic response was accompanied by an increase in intracellular ROS levels. Although ROS are often associated with oxidative damage, accumulating evidence indicates that tightly regulated ROS production acts as a physiological signaling mechanism during tissue repair [42,43]. Specifically, moderate ROS levels regulate fibroblast proliferation, migration, angiogenesis, and extracellular matrix remodeling, whereas excessive or persistent ROS accumulation results in oxidative stress and impaired wound healing. This interpretation is consistent with our previous findings in equine dermal fibroblasts, where A-PRF+ induced a ROS-dependent metabolically active phenotype associated with increased cellular organelle activity, including processes such as increased protein synthesis and trafficking through the endoplasmic reticulum, as well as lipid metabolism for membrane remodeling [44,45,46].

The metabolic activation induced by A-PRF+ was subsequently translated into functional changes that became evident in the longer-term assays. ECSCs exposed to A-PRF+ exhibited accelerated wound closure and enhanced cell cycle re-entry compared with serum-starved control cells. In all experiments, 10% FBS was included as a positive control; however, in the wound healing assay, serum supplementation contributes to wound closure through both proliferative and migratory effects [47]. Conversely, A-PRF+-mediated wound closure was observed in the absence of exogenous serum and likely reflects the combined contribution of cell migration and proliferation, consistent with the increased cell cycle re-entry observed following A-PRF+ treatment. Together, these findings suggest that the initial metabolic and redox responses evolved into a sustained pro-regenerative phenotype rather than representing a transient metabolic adaptation.

Corneal fibroblasts are essential mediators of tissue repair. Following corneal injury, quiescent keratocytes become activated, acquire a fibroblastic phenotype, migrate into the wounded area, proliferate, and synthesize extracellular matrix components required for stromal regeneration. This coordinated response is fundamental for restoring corneal integrity, although its dysregulation may also contribute to fibrosis and scar formation. Wilson et al. [48] extensively described these cellular events as key phases of corneal wound healing, highlighting fibroblast activation as a critical determinant of tissue repair.

The accelerated wound closure observed in the present study is therefore consistent with the established biological role of platelet-derived growth factors in promoting fibroblast responses involved in tissue repair, including both migration and proliferation [49,50]. Platelet concentrates, including platelet-rich fibrin, continuously release bioactive molecules such as PDGF, TGF-β, VEGF, EGF, and IGF-1, which activate intracellular signaling pathways involved in cytoskeletal and membrane remodeling, proliferation, and migration [51,52,53,54]. This sustained release has been proposed as one of the major advantages of platelet-rich fibrin over first-generation platelet concentrates [55]. Importantly, given the 72 h duration of the wound healing assay, the observed wound closure likely reflects the combined contribution of cell migration and proliferation, consistent with the increased cell cycle re-entry observed following A-PRF+ treatment. However, these effects should not be interpreted exclusively as evidence of a pro-reparative response, since fibroblast activation is a shared component of both physiological stromal repair and fibrotic wound healing. Therefore, the increased collagen production and fibroblast-mediated wound closure observed in our experimental model should be interpreted as evidence of processes occurring within the broader wound-healing response, rather than as definitive evidence of corneal regeneration.

The use of A-PRF+ offers several advantages over other platelet concentrates. Unlike PRP, PRF is prepared without anticoagulants or exogenous clotting agents, allowing natural fibrin polymerization [16,56]. When prepared, as in the present study, using additive-free glass or certified additive-free plastic tubes, PRF also avoids potential contamination by silica microparticles that may be released from silica-coated blood collection tubes [57]. A disadvantage is that both PRF clots and injectable PRF (I-PRF), the liquid form of PRF, must be used immediately after preparation, as I-PRF remains fluid for only a short period (approximately 15–30 min) before fibrin polymerization occurs [58]. This represents a practical limitation for ophthalmic applications, where topical therapies generally require repeated administration several times daily over prolonged treatment periods [59]. In this context, the use of A-PRF+-derived conditioned medium may represent a practical alternative, allowing the administration of a standardized preparation enriched in bioactive growth factors while avoiding the handling limitations associated with fresh PRF.

This hypothesis is supported by previous in vitro studies. Human-derived PRF obtained using different centrifugation protocols, when used either as a scaffold or as conditioned medium for limbal explant cultures, released significantly higher concentrations of growth factors and promoted greater cell proliferation, migration, viability, and maintenance of stemness than conventional cultures on amniotic membranes [60].

However, conflicting results have been reported regarding the use of PRF for corneal healing. Choi et al. [61] did not recommend PRF for the treatment of experimentally induced corneal wounds in rabbits because it was less effective than PRP. The authors suggested that the high fibrin content, variable platelet concentration, and abundant leukocyte population within PRF may trigger excessive inflammatory responses. Similarly, serum eye drops obtained by mechanical agitation of PRF clots and therefore containing high concentrations of fibrin-derived components were associated with corneal opacity, vascularization, melanosis, and other adverse outcomes during the treatment of corneal ulcers in donkeys, limiting the potential therapeutic benefits of this PRF formulation [10].

In contrast, other studies have reported favorable results with different PRF formulations. A-PRF gel promoted epithelial and stromal regeneration in experimentally induced corneal ulcers in donkeys, while the combined use of solid and liquid horizontally centrifuged PRF showed beneficial effects in horses [9,11]. These apparently contradictory findings may reflect differences in PRF preparation protocols and composition. Although the fibrin scaffold of PRF membranes provides sustained release of growth factors [14,62], its direct application to the ocular surface may interfere with corneal epithelial healing because the fibrin matrix acts as a temporary biomaterial that may influence cell migration and stromal remodeling [61]. Therefore, removal of the fibrin scaffold while preserving the released growth factors, as achieved with an A-PRF+-conditioned medium, may retain the biological activity of PRF while avoiding the presence of a fibrin matrix on the corneal surface.

Importantly, the responses observed in fibroblasts derived from the two donors, despite their markedly different ages, were consistent, supporting the reproducibility of the observed effects within the exploratory scope of the present study. However, the limited number of biological donors does not allow conclusions to be drawn regarding age-dependent responses or broader inter-individual variability. Furthermore, the limited number of biological donors represents a limitation of the present study; however, this sample size should be considered within the exploratory nature of this first investigation of an A-PRF+-conditioned medium in equine corneal fibroblasts. In addition, the two donors represented markedly different age groups, and the present design does not allow the contribution of donor age to be specifically assessed. Although the responses observed in the two donors were consistent, future studies including a larger number of donors distributed across different age groups would be valuable to better characterize inter-individual variability and the potential influence of donor age on the cellular response to A-PRF+.

Additionally, although further investigation is required to confirm its therapeutic efficacy and to determine whether these responses translate into effective stromal repair without promoting fibrotic remodeling and scar formation, with consequent effects on corneal transparency, these results support the A-PRF+-conditioned medium as a promising and minimally manipulated approach for enhancing fibroblast-mediated wound-healing responses in the horse. However, whether these effects translate into effective corneal regeneration while preserving normal stromal architecture remains to be confirmed in vivo.

## 5. Conclusions

The present study demonstrates that a conditioned medium derived from allogeneic A-PRF+ promotes a specific biochemical response in primary equine corneal fibroblasts, characterized by early metabolic activation, controlled ROS generation, enhanced wound closure, cell cycle re-entry, and increased growth factor release and collagen type I expression. These findings extend previous observations obtained in equine dermal fibroblasts and indicate that the biological activity of A-PRF+ is not limited to a specific tissue source but can also influence clinically relevant ECSCs.

The ability of A-PRF+ soluble factors to stimulate fibroblast functions in the absence of an exogenous serum highlights its potential as a biological support for corneal repair, particularly by promoting cell migration and regenerative responses during the early phases of wound healing. The sustained release of platelet-derived bioactive molecules may represent an important mechanism underlying these effects. However, further investigations are needed to establish the clinical relevance of these findings and assess whether A-PRF+ supports tissue recovery while preserving stromal architecture and optical clarity, without inducing excessive fibrosis or persistent scar formation.

Moreover, this study provides novel mechanistic insights into the early cellular responses induced by A-PRF+ in equine corneal fibroblasts, contributing to the limited understanding of the cellular mechanisms underlying the biological effects of platelet-rich fibrin in corneal repair.

Further in vitro and in vivo studies are warranted to compare the biological effects of the different solid and liquid PRF formulations. Such studies should characterize growth factor release, platelet and fibrin content, and evaluate different concentrations and dosing regimens on the various cell populations involved in the complex process of corneal wound healing.

## Data Availability

The original contributions presented in this study are included in the article/Supplementary Materials. Further inquiries can be directed to the corresponding authors.

## Supplementary Materials

The following supporting information can be downloaded at https://www.mdpi.com/article/10.3390/ani16213341/s1. Figure S1: Representative fluorescence microscopy images of primary equine corneal fibroblasts stained with Calcein AM and propidium iodide (PI), after 24 h of treatment. The panel includes Calcein AM fluorescence (green, live cells), PI fluorescence (red, dead cells), and the merged image (green + red). Scale bar: 100 μm. N ≥ 5. Magnification: 20×. Figure S2: Representative fluorescence microscopy images of primary equine corneal fibroblasts stained with DCFH-DA to evaluate intracellular reactive oxygen species (ROS) after 24 h of treatment. The panel includes brightfield (cell morphology), DCFH-DA fluorescence (ROS; green), and the merged image (brightfield + green fluorescence). Scale bar: 100 μm. N ≥ 5. Magnification: 20×. Figure S3: Cell metabolic activity was assessed by the MTT assay after 72 h of treatment. Data are presented as mean ± standard deviation (SD) of three independent experiments. *** *p* < 0.001 vs. Ctrl. Table S1: Baseline growth factor concentrations in 1:4 diluted A-PRF+ conditioned medium. Data are presented as mean ± standard deviation (SD) of three experimental replicates.

## Abbreviations

The following abbreviations are used in this manuscript:

A-PRF+	Advanced Platelet-Rich Fibrin Plus
ARRIVE	Animal Research: Reporting of In Vivo Experiments
ANOVA	Analysis of Variance
BSA	Bovine Serum Albumin
Calcein-AM	Calcein Acetoxymethyl Ester
Ctrl	Control
DCFH-DA	2′,7′-Dichlorodihydrofluorescein Diacetate
DMEM	Dulbecco’s Modified Eagle Medium
DMSO	Dimethyl Sulfoxide
DNA	Deoxyribonucleic Acid
ECSC	Equine Corneal Stromal Cells
EDTA	Ethylenediaminetetraacetic Acid
EGF	Epidermal Growth Factor
ELISA	Enzyme-Linked Immunosorbent Assay
FBS	Fetal Bovine Serum
G0/G1	Gap 0/Gap 1 Phase
G2	Gap 2 Phase
IGF-1	Insulin-Like Growth Factor 1
MTT	3-(4,5-Dimethylthiazol-2-yl)-2,5-diphenyltetrazolium bromide
PBS	Phosphate-Buffered Saline
PDGF	Platelet-Derived Growth Factor
PDGF-BB	Platelet-Derived Growth Factor-BB
PI	Propidium Iodide
PRF	Platelet-Rich Fibrin
PRGF	Plasma Rich in Growth Factors
PRP	Platelet-Rich Plasma
ROS	Reactive Oxygen Species
S	Synthesis Phase
SD	Standard Deviation
s-PRF	Standard Platelet-Rich Fibrin
TGF-β	Transforming Growth Factor Beta
TGF-β1	Transforming Growth Factor Beta 1
VEGF	Vascular Endothelial Growth Factor
VEGF-A	Vascular Endothelial Growth Factor A
