# Supplementary material for: Biological Effects of Advanced Platelet-Rich Fibrin+ (A-PRF+)-Conditioned Medium on Primary Equine Corneal Stromal Cells: A Proof-of-Concept Study
Source: Animals (Basel). 2026 Oct 23;16(21):3341. doi: 10.3390/ani16213341 (PMC13761169; doi:10.3390/ani16213341)
Supplement: Supplementary file 1 [file animals-16-03341-s001.zip › animals-4651836-supplementary.pdf]

Supplementary Materials

Biological Effects of Advanced Platelet-Rich Fibrin+ (A-PRF+) Conditioned Medium on Primary Equine Corneal Stromal Cells: A Proof-of-Concept Study

Maria Rosaria Miranda [1,2], Rodolfo Maria Abbate [1], Mariaelena de Chiara [3], Barbara Lamagna [3], Chiara Del Prete [4], Giuseppe Piegari [4], Davide De Biase [1], Chiara Montano [5], Alessia Bertamino [1], Pietro Campiglia [1], Maria Pia Pasolini, [3,*,‡] and Vincenzo Vestuto [1,*,‡]

[1] Department of Pharmacy, University of Salerno, 84084, Fisciano, Italy
[2] Department of Medicine, Case Western Reserve University School of Medicine, Cleveland, OH 44106
[3] Department of Veterinary Medicine and Animal Production, University of Naples Federico II, Naples, Italy
[4] Department of Human Sciences, Link Campus University, Roma, 00165, Italy
[5] San Rocco Factory, via Montirone 18, 40019 Bologna, Italy
* Correspondence: pasolini@unina.it, vvestuto@unisa.it
‡ These authors equally contributed to the work.

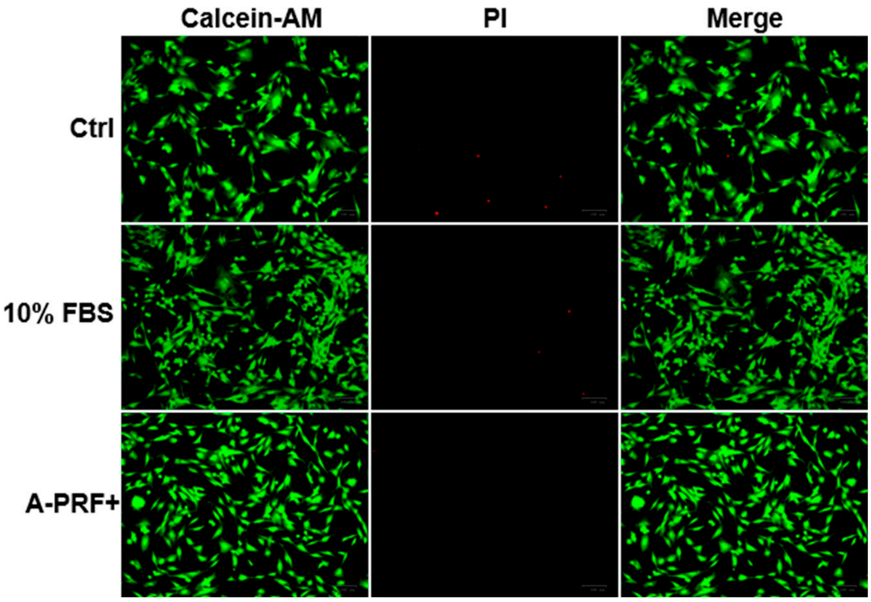

Figure S1. Representative fluorescence microscopy images of primary equine corneal fibroblasts stained with Calcein AM and propidium iodide (PI), after 24 h of treatment. The panel includes Calcein AM fluorescence (green, live cells), PI fluorescence (red, dead cells), and the merged image (green + red). Scale bar: 100 μm. N ≥ 5. Magnification: 20×.

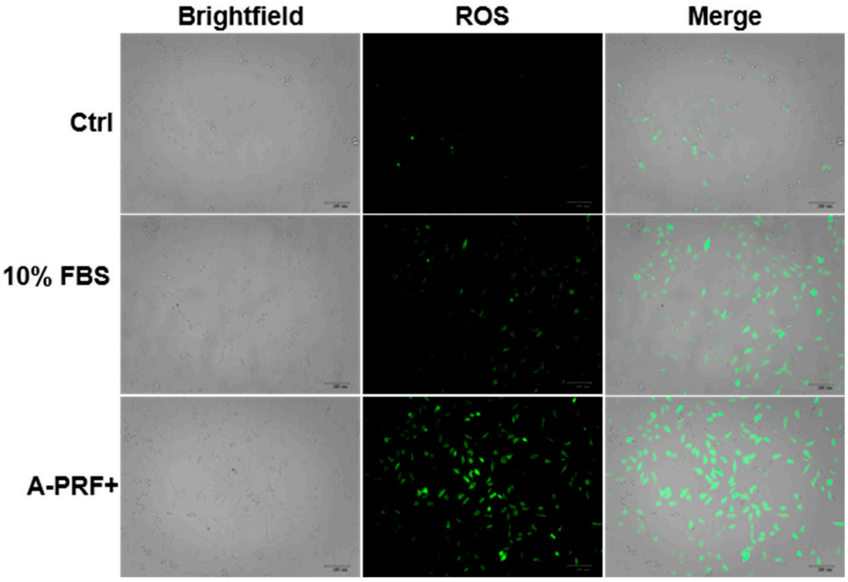

Figure S2. Representative fluorescence microscopy images of primary equine corneal fibroblasts stained with DCFH-DA to evaluate intracellular reactive oxygen species (ROS) after 24 h of treatment. The panel includes brightfield (cell morphology), DCFH-DA fluorescence (ROS; green), and the merged image (brightfield + green fluorescence). Scale bar: 100 μm. N ≥ 5. Magnification: 20×.

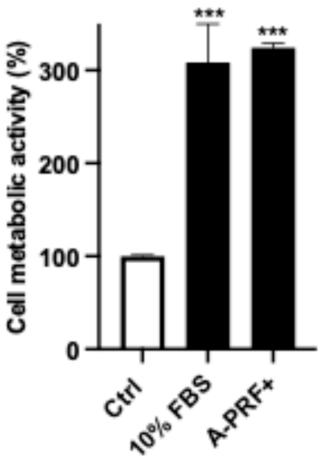

Figure S3. Cell metabolic activity was assessed by the MTT assay after 72 h of treatment. Data are presented as mean ± standard deviation (SD) of three independent experiments. *** $p < 0.001$ vs. Ctrl.

Table S1. Baseline growth factor concentrations in 1:4 diluted A-PRF+ conditioned medium. Data are presented as mean ± standard deviation (SD) of three experimental replicates.

**Growth factor**	**A-PRF+ 1:4 baseline (pg/mL ± SD)**	**Statistical comparison vs. A-PRF+-treated ECSC**
PDGF-BB	139.1 ± 9.37	$p < 0.001$
TGF-β1	3.25 ± 0.1	$p < 0.001$
VEGF-A	1.7 ± 0.1	$p < 0.001$
